# Source analysis of P3a and P3b components to investigate interaction of depression and anxiety in attentional systems

**DOI:** 10.1038/srep17138

**Published:** 2015-11-24

**Authors:** Yuezhi Li, Wuyi Wang, Tiebang Liu, Lijie Ren, Yunfei Zhou, Changhong Yu, Xingda Qu, Yong Hu

**Affiliations:** 1 Laboratory of Neural Engineering, Shenzhen University, Shenzhen, China, 518060; 2 Department of Orthopaedics and Traumatology, The University of Hong Kong, Pokfulam, Hong Kong; 3 Department of Psychological and Brain Sciences, University of California, Santa Barbara, Santa Barbara, CA, 93106, USA; 4 Shenzhen Kangning Hospital, Shenzhen, China, 518020; 5 The first Affiliated Hospital of Shenzhen University, Shenzhen, China, 518035

## Abstract

This study examined the impact of depressive disorders, anxiety disorders and the comorbidity of these disorders on the regional electrophysiological features of brain activity. Sixty-four-channel event-related potentials (ERP) were acquired during a visual oddball task in patients with depressive disorder, patients with anxiety disorders, patients with comorbid depressive and anxiety disorders and healthy subjects. An fMRI-constrained source model was applied to ERP to identify different cortical activities in the patient and control groups. Comorbid patients showed an abnormal frontal-greater-than-parietal P3b topography in the right hemisphere and the highest P3a amplitude at frontal and central sites at the scalp midline. For P3b, depressed patients showed decreased right-lateralized activity in the precentral sulcus (PrCS) and posterior parietal cortex (PPC). Anxious patients demonstrated hyperactive prefrontal cortices (PFC). Comorbid patients presented decreased activity in the cingulate gyrus, right PrCS and right PPC and increased activity in the left PFC and left insular (INS). For P3a, hyperactive left PrCS was found in comorbid patients. Comorbid patients showed both anxiety-related and depression-related activity. A superimposition effect of depression and anxiety was identified with (1) aggravated hypo-function of the right-lateralized dorsal attention and salience networks and (2) complicated anxiety-related hyper-function of the left-lateralized ventral attention and salience networks.

Depression and anxiety disorders are frequently comorbid, which makes their diagnosis and treatment difficult^1^,^2^,^3^,^4^,^5^. Few studies have examined the neurobiology of anxious depression, and it is important to expand our knowledge about the comorbidity of depression and anxiety, which will promote strategies for the diagnosis and treatment of this comorbid illness.

Investigations of functional network dysfunction have revolutionized our understanding of psychiatric disorders from symptom-based classification to network-level pathology^6^. For example, anxiety disorders are associated with increased activity in the ventral attention network and cingulo-opercular network (also referred to as the salience network)^7^,^8^,^9^ and decreased activity the fronto-parietal network (referred to as the executive control network)^10^,^11^. In contrast, major depression is associated with increased activity in the cingulo-opercular network and decreased activity in the fronto-parietal network^12^,^13^,^14^,^15^. Davidson^16^ has proposed the approach-withdrawal theoretical framework, which hypothesizes two separate systems for emotion and motivation that may function abnormally in anxiety and depressive disorders. The approach system controls behavioral motivation toward reward and implicates the left frontal lobe regions, which are hypothesized to be hypoactive in depression. The withdrawal system controls behavioral inhibition and implicates the right frontal lobe regions, which are hypothesized to be hyperactive in anxiety. In addition, Heller has proposed the valence-arousal model^17^ and hypothesized that depression correlates with decreased activity in the right parieto-temporal brain region associated with arousal properties and that anxiety correlates with increased activity in this region. Although there have been network-based studies of depression and anxiety, the functional networks related to the comorbidity and interaction of anxiety and depression remain unexplored. These networks are investigated in the present study.

Because attentional processes can be altered by depression and anxiety, the present study used the well-established oddball paradigm for target and distracter processing to reveal activated brain networks^18^,^19^,^20^. The anxiety-related and depression-related patterns during attentional processes were investigated. The P3b potential elicited by target stimuli has been extensively studied in patients with depressive disorders. Although there have been conflicting findings, patients with depressive disorders tend to show some reduction of P300 to target stimuli in oddball tasks, especially in challenging oddball tasks^21^. Similarly, P3a potential may be decreased in patients with depressive disorders^22^. There are few P300 studies of patients with anxiety disorders, and auditory oddball stimuli were usually used in these studies. The results of the previous P300 studies of anxiety disorders have not suggested consistent patterns. Decreased parietal P3b in patients with anxiety disorders has been reported in a few studies^23^,^24^,^25^, whereas increased frontal P300 subcomponent has been found to be associated with anxiety disorders in one study^26^. Additionally, few studies have investigated P300 in comorbid patients. A previous study has reported an increase in late P300 subcomponents with regard to the comorbidity of depression and anxiety^26^. Based on the above findings, we expected to find decreased parietal P3b and increased frontal P3b in the comorbid patients in the present study.

Investigating the alteration of P300 topography and source activity provided opportunities to better understand attentional deficits in psychiatric patients. We examined the spatiotemporal dynamics of brain networks during attentional processes with regional source analyses of 64-channel event-related potential (ERP) recordings, and the sources were separately seeded at the foci of the main fMRI activity clusters in identical tasks^27^. Regional source activity that accounts for scalp ERP data is used to estimate the magnitude, orientation and time course of local current vectors. Regional source calculations divide local cortical activity into three mutually orthogonal components, which makes it beneficial to analyze local current flow variations in psychiatric disorders in any arbitrary direction. More importantly, both the classical parietal P300 component, or P3b, which occurs 300–600 msec after a target stimulus and has been linked to the cognitive processes of context updating, context closure, and event-categorization^28^,^29^, and the slightly earlier fronto-central P3a, which has mainly been associated with orienting responses, can be elicited in the three-stimulus oddball paradigm. Therefore, comparisons of the cortex generation of P300 components (i.e., P3a and P3b in the present study) between depression and anxiety patients are well suited for addressing the issue of attentional systems in psychiatric patients.

## Methods

### Participants

This study was performed in accordance with relevant guidelines and regulations approved by the institutional review board of Shenzhen University. All subjects provided informed consent before participating in this study. Ninety-five subjects were recruited into four groups (Table 1): (1) HC: healthy controls; (2) D-alone: depressive disorder without anxiety disorder; (3) A-alone: anxiety disorders without depressive disorder; and (4) D + A: depressive disorder plus co-existing Axis I anxiety disorder^2^,^30^,^31^. The healthy control subjects were recruited from the hospital or university staff and were then screened by psychiatrists. Patients in the other three groups were referred by psychiatrists in the Depression and Anxiety Disorders Clinic in Shenzhen Kangning Hospital, and the diagnoses were made by two senior psychiatrists with more than 10 years’ experience, based on Structured Clinical Interview for DSM- IV Axis I Disorders, Clinician Version (SCID-CV)^32^. Patients in Group 2 met the DSM-IV criteria for major depressive disorder (n = 21) or dysthymia (n = 3), but not for any anxiety disorder. The patients in Group 3 met the DSM-IV criteria for one or two anxiety disorders, but not for depressive disorder, and were diagnosed with generalized anxiety disorder (n = 14), panic disorder (n = 6), social phobia (n = 5) or a combination of these disorders (n = 3). The patients in Group 4 met the DSM-IV criteria for major depressive disorder (n = 18) or dysthymia (n = 2) and met the DSM-IV criteria for generalized anxiety disorder (n = 12), panic disorder (n = 5) or social phobia (n = 3). Subjects with other neurological disorders, a history of head trauma, or who had taken drugs in the four weeks before the study, were excluded. From May 1 to June 30, 2012, a consecutive series of 275 outpatients in the Depression and Anxiety Disorders Clinic were referred based on the inclusion criteria. 209 patients were excluded because of other neurological disorders or history (39 cases), taking medication in the study period (84 cases) and rejection of informed consent (86 cases).

### Experimental procedure

A visual three-stimulus oddball task that has been reported in previous studies was used^19^,^27^. To control for lower-level visual attributes, which are a common confound of the classical version of the three-stimulus oddball task with novel and/or complex figures, two different task types (circle task and square task; Table 2) were used, in which simple stimulus features were counter-balanced. The stimuli were solid blue shapes presented in a random series, once every 2 sec, for 75 msec. Each of the tasks was presented in three runs with 200 stimuli each. The order of the two task types was counterbalanced across participants. The stimuli were defined as target, distracter, and standard and were presented with the probabilities of 0.05, 0.05, and 0.90, respectively. The target and standard stimuli differed in their viewing angles, and the distracter stimulus was a circle in square task and a square in circle task, respectively. The task was to respond to the target stimuli by pushing a mouse button with the right thumb as quickly and correctly as possible. For misses (absence of button press after target stimulus) and false alarms (button press after standard or distracter stimulus), the corresponding event was excluded from the analysis. Before each task, all subjects underwent a practice block of 50 stimuli (20 targets, 30 standards).

### EEG recording and analyzing

Electroencephalogram (EEG) data were continuously recorded (band pass 0.05–100 Hz, sampling rate 1000 Hz) with a NeuroScan system (NeuroScan Lab, Charlotte, NC, USA), using an electrode cap with 64 Ag/AgCl electrodes. The electrodes were placed according to the 10–20 System (Fp1, Fp2, F7, F3, Fz, F4, F8, T7, C3, Cz, C4, T8, P7, P3, Pz, P4, P8, O1, Oz, and O2); additional intermediate sites were AF3, AF4,Fpz, F1, F2, F5, F6, FC1, FC2, FC3, FC4, FC5, FC6, FCz, FT7, FT8, C1, C2, C5, C6, CP1, CP2, CP3, CP4, CP5, CP6, CPz, TP7, TP8, P1, P2, P5, P6, M1, M2, PO3, PO4, PO5, PO6, PO7, PO8, POz, CB1,CB2. All channels were referenced to the tip of the nose (recalculated off-line against the average reference). VEOG and HEOG were recorded with two pairs of electrodes: one was placed above and below the right eye, and the other was placed 10 mm from the lateral canthi. Electrode impedance was maintained below 5 kΩ throughout the experiment.

EOG artifacts were corrected using automated ocular artefact reduction module in Scan (NeuroScan Lab). The EEG was digitally filtered with a low-pass filter at 30 Hz (24 dB/Octave) and segmented in epochs of 1200 ms, time-locked to stimulus onset and including a 200 ms pre-stimulus baseline. Trials contaminated by amplifier clipping, bursts of EMG activity, or peak-to-peak deflection exceeding ±100 μV were excluded from averaging. The number of accepted trials was 785.5 ± 18.3, 47.6 ± 2.7 and 45.8 ± 2.3 for standard, target, and distracter stimuli, respectively. For the target event, the number of accepted trials differed across groups. It was smaller in the comorbid patients (46.1 ± 2.7) than in the anxiety group (48.0 ± 2.5) and controls (48.2 ± 2.2) (ps < 0.05). For the standard and distracter events, the number of accepted trials did not differ significantly across groups (ps > 0.05).

The ERPs were computed separately for the target, distracter, and standard conditions. Difference waveforms were calculated by subtracting the ERP of the standard stimuli from that of the targets and distracters, respectively. The individual difference ERP waves were exported into the standardized 81 electrode configuration of the 10-10 system by Brain Electrical Source Analysis software (BESA) and band-pass filtered at 0.1–15 Hz for source analysis. Grand average difference waves were obtained from the average of all participants. Topographical maps of scalp voltage were also produced at the respective peak latencies of the ERP in the target and distracter conditions.

The P3a (distracter effect) and P3b (target effect) components were defined as the largest positive deflection within the time window between 300 and 600  msec. Peak latency was defined as the time from stimulus onset to the peak of each scalp component. The measurements were submitted to a four-way repeated-measure ANOVA with stimulus types (target and distracter), electrode sites (F3/4/z, C3/4/z, P3/4/z), and hemisphere (left, midline, right) as within-subject factors and group (HC, D-alone, A-alone and D + A) as the between-subjects factor. Greenhouse–Geisser correction was used, and corrected p values were reported. All data were expressed as the means ± standard error.

### ERP source analysis

The recorded difference ERP waves were imported into BESA from a standardized 81 electrode set of the 10-10 system. The source activity was calculated within a four-shell spherical head model. A previously reported regional source (RS) model defined by fMRI activity was used as a common model for all subjects^27^ (Table 3). This model was then applied to the grand average difference ERP waves to fit the three orientations of each RS for the target and distracter conditions: (1) the first orientation of the RS was set to match the main current flow direction of the grand average difference ERP waves; (2) the second orientation was automatically set to model the largest current flow perpendicular to the first orientation; and (3) the third orientation was set to remain mutually orthogonal.

After the common source model was obtained, it was applied to individual ERP data, and the individual source activities were estimated according to the best correspondence between the recorded and estimated scalp distribution^33^. The correspondence was evaluated by calculating the residual variance of signals. The individual source activities were averaged across subjects to obtain the grand average source activity for each group in each condition. The direction of current flow of the source activities was projected back to the scalp voltage to calculate topographical maps at the respective latencies.

### Statistical analysis

Fourteen RSs and three orthogonal dipoles per RS, for a total of 42 channels of source activity for each condition, were analyzed in BESA Statistics. A two-step analysis was conducted.

In the first step, to identify significant difference between groups, two-tailed t-tests were performed per data point to retrieve preliminary significant effects between the two groups. The data for t-tests were taken at 0–1000 ms post-stimulus. Clustering in time of the preliminary significant effects was performed depending on a cluster alpha setting of 0.05 and adjacency of data-points. The cluster value was derived from the sum of t-values of all data points in the cluster. Then, data clusters were used for a permutation test to examine the significance of effects. The permutation was executed 2000 times. In the permutation, the data of subjects are interchanged. For all calculated permutations, a new distribution of cluster value is determined. Based on this distribution, the significance of the initial cluster value can be determined, and the p-values resulting from the permutation test were corrected for multiple comparisons. Depending on the direction of statistical effects, positive and negative clusters could be identified.

In the second step, if both positive and negative clusters with p-value less than 0.1 were identified, they would be transformed into the same direction (e.g. all positive) by inverting dipolar orientation in BESA. Then one-tailed t-tests, clustering and permutation test were performed to help obtain the final results about significant clusters. If clusters with p-value less than 0.1 having the same direction were identified, one-tailed t-test, clustering and permutation test were directly performed to determine significant clusters.

Because there are six such comparisons by BESA Statistics from four participant groups, we used the Holm-Bonferroni method to control the family-wise error rate^34^ (see Supplemental Figure S1 online).

## Results

### Behavioral data

The behavioral results are summarized in Table 4. For reaction time (RT) analysis, there was a significant main effect of group (F(3,91) = 3.81, p = 0.013, partial η^2^ = 0.231), which indicates that D + A pressed the button more slowly than did HC (p = 0.023), whereas the response speed did not differ between HC and A-alone or D-alone groups (ps > 0.05). In comparison with HC, D + A showed a reduced hit rate for target stimulus (p = 0.005) and an increased false positive rate for the standard stimulus (p = 0.023). No other effects were significant (ps > 0.1).

### ERPs analysis

**Figure 1:**
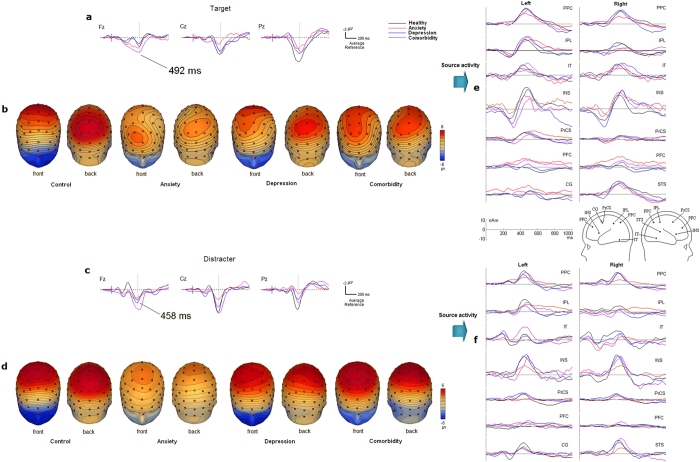
ERP waveforms, topographic ERP maps and source activities in target and distracter conditions during visual oddball paradigm. (**a**) Grand average difference ERP waves for the target stimuli at typical midline electrodes (filtered at 0.1–15 Hz); (**b**) Topographical map of scalp voltage for the target P300 for all groups; (**c**) Grand average difference ERP waves for the distracter stimuli at typical midline electrodes (filtered at 0.1–15 Hz); (**d**) Topographical map of scalp voltage for the distracter P300 for all groups. (**e**) Grand average source activity of the main current flow direction (also referred as orientation 1) of each RS in target condition. (**f**) Grand average source activity of the main current flow direction of each RS in distracter condition. Position of RSs is indicated on BESA standard head model.

Figure 1a–d shows the grand averaged difference ERP waves and topographical maps of scalp voltage for P3a and P3b elicited by the target and distracter conditions, respectively. The 4-way ANOVA for P3 amplitudes revealed that overall, the main effect of group was significant (F(3,91) = 5.549, p = 0.002, partial η2 = 0.447). Further analysis showed that the mean value of the P3 amplitude was smaller in A-alone group (1.935 ± 0.176 μV) than in HC (3.140 ± 0.133 μV, p = 0.001), D-alone (2.998 ± 0.169 μV, p = 0.006) and D + A group (3.406 ± 0.249 μV, p = 0.002), respectively. There was no significant difference in the P3 amplitude among the latter three groups (p > 0.05).

The main effect of stimulus type was significant (F(1,91) = 10.739, p = 0.002, partial η2 = 0.358) and qualified by the two-way interaction of stimulus × electrode site (F(2,182) = 28.569, p < 0.001, partial η2 = 0.587), as well as the three-way interaction of stimulus type × electrode site × hemisphere (F(4,364) = 11.474, p < 0.001, partial η2 = 0.567). Post hoc analysis revealed that at the frontal sites, P3a elicited by distracter stimuli was larger (2.775 ± 0.178 μV) than P3b elicited by target stimuli (2.343 ± 0.302 μV; p > 0.05), whereas at parietal sites, P3b was larger (4.455 ± 0.221 μV) than P3a (1.543 ± 0.156 μV; p < 0.001); this effect was more conspicuous at left hemisphere (F(2, 182) = 43.822; p < 0.001).

We found a significant 4-way interaction of stimulus type × electrode site × hemisphere × group (F(12,364) = 2.374, p = 0.007). Therefore, separate three-way ANOVA of group, electrode site and hemisphere was conducted for P3a for distracter and P3b for target stimuli, respectively.

**Figure 2:**
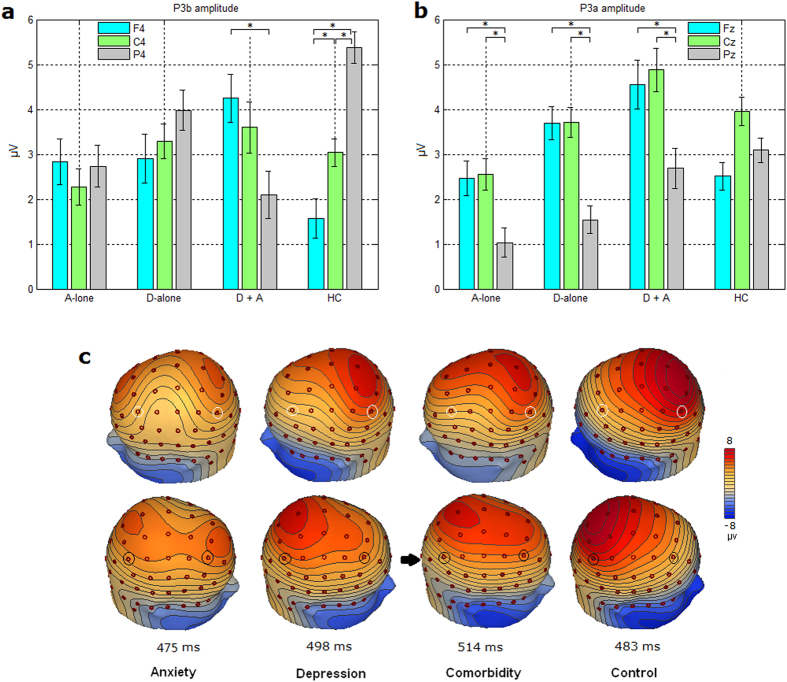
Alteration of P300 amplitude at frontal, central and parietal sites in patient groups. (**a**) Bar graph to show the effect of electrode site on P3b amplitude in the right hemisphere in participant groups. Amplitude was plotted as mean ± standard error. (**b**) Bar graph to show the effect of electrode site on P3a amplitude at the scalp midline in participant groups. Amplitude was plotted as mean ± standard error. (**c**) Altered scalp voltage maps of the P3b in psychiatric patients, particularly in the right hemisphere in the comorbid patients. Peak latencies of P3b are respectively indicated. The circled electrode sites are F4 and P4.

For P3b analysis, the ANOVA showed that both the two-way interaction of group × electrode site (F(6,182) = 3.239; p = 0.013) and the three-way interaction of group × electrode site × hemisphere (F(12,364) = 2.308, p = 0.015) were significant. Further analysis showed that the interaction of group × electrode site was more conspicuous in the right hemisphere (p = 0.004). Post hoc analysis revealed that for both the A-alone and D-alone groups, there was no significant effect of electrode site (ps > 0.05). For HC, the P3b amplitudes were larger at parietal site than at frontal site (p < 0.001). For D + A, P3b was smaller at parietal site than at frontal site (p = 0.03) (see Fig. 2a). Figure 2c shows increased frontal P3b and decreased parietal P3b in patient groups in the right hemisphere, particularly in the comorbid patients.

For P3a analysis, the ANOVA showed a significant three-way interaction of group × electrode site × hemisphere (F(12,364) = 2.123, p = 0.032), and post hoc analysis revealed that the interaction of group × electrode site was significant at the scalp midline (p = 0.022). For HC, there was no significant effect of electrode site (ps > 0.05), whereas for D-alone, P3a at Fz site and Cz site were larger than that at Pz site (p = 0.027 and p = 0.001); for A-alone, P3a at Fz site and Cz site were larger than that at Pz site (p = 0.009 and p = 0.001); and for D + A, P3a at Fz site and Cz site were larger than that at Pz site (p = 0.050 and p = 0.034) (see Fig. 2b). Among all of the groups, D + A showed the highest frontal and central P3a activities. All patient groups showed smaller P3a at the parietal site than did healthy controls. Additionally, the interaction of group × electrode site was not significant in the left and right hemispheres (ps > 0.05).

For the 4-way ANOVA analysis of P3 latency, we found a main effect of stimulus type only (F(1,91) = 6.528, p = 0.014, partial η2 = 0.453), and post hoc analysis revealed that P3a latency was shorter (459 ms) than P3b latency (492 ms; p = 0.014). No other main effects and interactions reached significance level (ps > 0.05).

### Source analysis

The location of fMRI-guided sources is depicted in Table 3 and Figure 1e,f. The bilateral parietal and temporal RSs and INS presented stronger activity in Figure 1e,f.

### Depression alone

**Figure 3:**
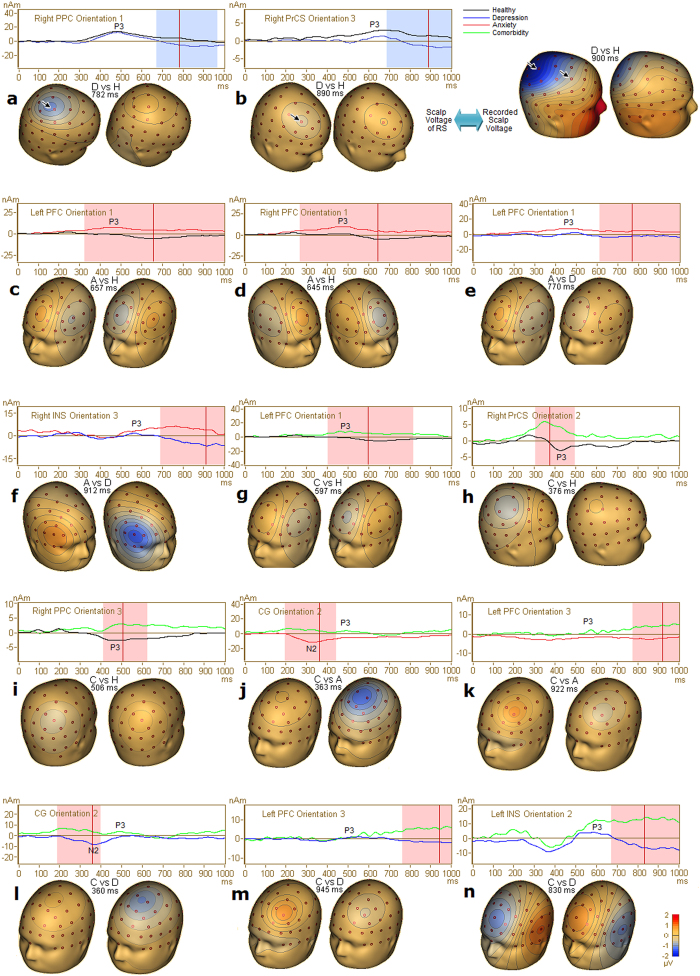
Significant clusters after Holm-Bonferroni method in target condition. For each plot of cluster, source activity and topographical maps of scalp voltage of the current flow direction of the corresponding RS were displayed and a mask indicates the significant time-period. Note that in the first row of maps, decreased source activities of the right PrCS and right PPC in the depression group in Figure 3a and Figure 3b showed good correspondence with depression-related alteration of topographical map. In the second row, increased source activities of the bilateral PFC in the anxiety group in Figure 3c and Figure 3d showed good correspondence with the anxiety-related alteration of topographical map at the frontal sites in Figure 1B. Abbreviation: (D), depression alone; (A), anxiety disorders alone; (C), comorbid patients; H, healthy subjects.

For the P3b component elicited by target stimulus, there was a significant difference between the depression and healthy groups in the right PPC and right PrCS during the late segment of the P3b epoch (Table 5). This showed negative foci over the right frontoparietal scalp area in the depression group compared with positive foci in healthy group (Fig. 3a,b). There was a significant difference between the depression and anxiety group in the right INS during the late segment of the P3b (Table 5), which demonstrated negative lateral frontal focus in the depression group, in contrast to the positive focus in the anxiety group (Fig. 3f).

**Figure 4:**
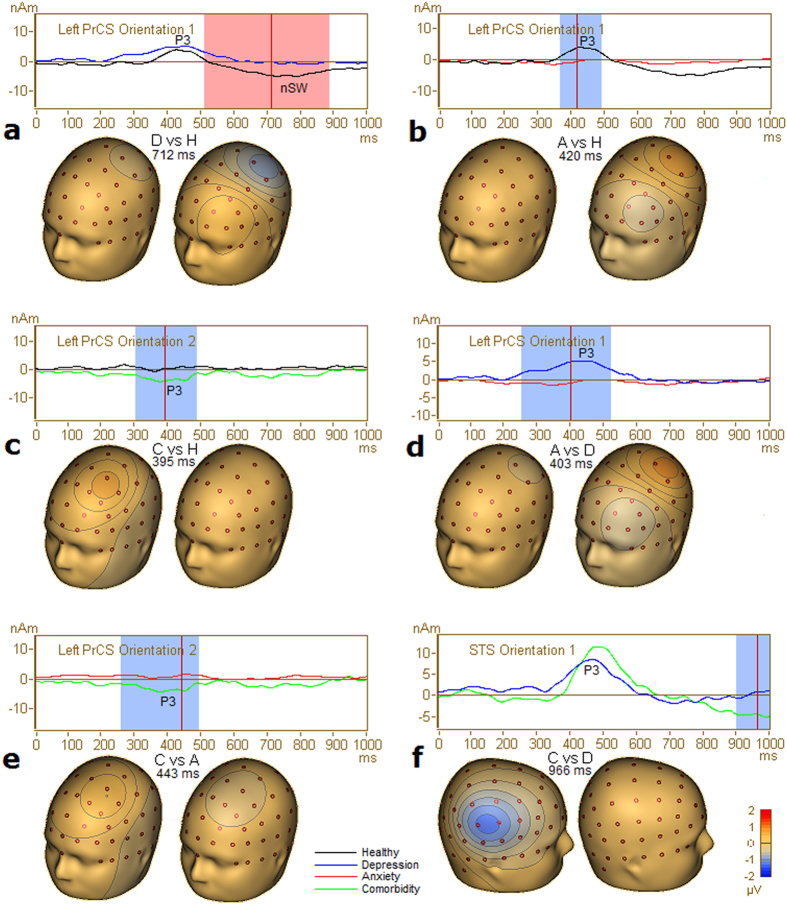
Significant clusters after Holm-Bonferroni method in distracter condition. Source activity and topographical maps of scalp voltage of the current flow direction of the corresponding RS were displayed and a mask indicates the significant time-period. Abbreviation: (D), depression alone; (A), anxiety disorders alone; C, comorbid patients; H, healthy subjects.

For the ERP component elicited by the distracter stimulus, there was a significant difference in the negative slow wave (nSW) following the P3a between the healthy controls and depression group in the left PrCS (Table 5; Fig. 4a). The depression group showed near-zero potential, whereas controls showed negative focus over the left central scalp area.

### Anxiety alone

For the P3b component, the activities in the left and right PFC were significantly different between the anxiety and healthy control groups (Table 5). Compared with the healthy group, the anxiety group exhibited positive foci with higher amplitude and longer duration over the bilateral medial prefrontal areas (Fig. 3c,d). A similar significant difference in the left PFC was found between the anxiety and depression groups (Fig. 3e; Table 5).

For the P3a component, the left PrCS demonstrated a significant difference between the anxiety group and the healthy or depression groups (Table 5; Fig. 4b,d). A near-zero potential over the left central area was observed in the anxiety group compared with a positive focus in the other two groups.

### Comorbidity

For the P3b component, there was a significant difference between the comorbid and healthy control groups in the left PFC, right PrCS and right PPC (Table 5), with foci of opposite polarity over the left prefrontal scalp area and the right frontoparietal areas (Fig. 3g–i). Interestingly, activity in the CG during the N200 period was significantly different between the comorbid and depression groups, as was the activity in the left PFC and left INS during the late segment of the P3b (Table 5). Topographical maps showed positive foci over the left medial frontal, frontal and operculum areas in the comorbid group compared with negative foci in the depression group (Fig. 3l–n). Activity in the CG during the N200 period was significantly different between the comorbid and anxiety groups (Table 5), as was activity in the left PFC during the late segment of the P3b. These activities showed negative foci over the medial frontal and frontal areas in the anxiety group compared with positive foci in the comorbid group (Fig. 3j,k).

For the P3a component, the second orientation of the left PrCS was significantly different between the comorbid group and the healthy control or anxiety groups (Table 5). The comorbid patients presented a positive focus over the left frontal area, whereas the anxiety or healthy groups showed a near-zero potential (Fig. 4c,e). Additionally, the right STS was significantly different between the comorbid and depression groups for P3a (Fig. 4f).

## Discussion

In comparison with healthy subjects, decreased P3b amplitude at parietal site and increased P3b amplitude at frontal site were found in the right hemisphere during a visual oddball task for all patients, but such effects were most evident in the comorbid patients. Furthermore, we identified network hypofunction in patients with depressive disorders alone, and network hyperfunction in patients with anxiety disorders alone. It was also found that aggravated network hypofunction and complicated network hyperfunction were associated with depression and anxiety comorbidity. This provides evidence of a superimposition effect of depression and anxiety in comorbidity, which facilitates better understanding of the mechanisms of cognitive deficit.

### Abnormality of P300 scalp potential

To extract features in P300 scalp potential, a separation of P3a and P3b analysis was carried out in this study. A significant interaction of electrode site and group in the right hemisphere was identified for P3b. Healthy subjects presented a parietal-greater-than-frontal P3b topography, whereas the comorbid patients showed an opposite frontal-greater-than-parietal P3b topography. This indicates increased activity at the frontal site and decreased activity at the parietal site in this hemisphere. According to the source analysis results, the reduction of the right parietal P3b in the comorbid patients was due to decreased activity of the right PPC and right PrCS.

Although there was no significant frontal-greater-than-parietal P3b topography in the right hemisphere in the depression group or anxiety group, decreased parietal P3b and increased frontal P3b were observed for both patient groups. Decreased parietal P3b with regard to depression and anxiety is consistent with previous studies^21^,^23^,^24^,^25^,^35^,^36^. Increased frontal P3b in the anxiety group arose from increased PFC activities according to the source analysis results. This finding is consistent with the increased frontal P300 subcomponent reported in a previous auditory oddball study^26^.

A significant interaction of electrode site and group at the scalp midline was identified for P3a. All patient groups showed a greater P3a at the frontal and central sites than at the parietal site, which was not seen in healthy subjects. Among all groups, the comorbid patients showed the highest frontal and central P3a activities, which might be associated with the increased activity of the left PrCS according to the source analysis.

### Source characteristics of anxiety disorders alone

Anxious patients are thought to show increased activity in the ventral attention network during stimulus-driven attention^37^,^38^. Increased fMRI activity in the ventral PFC has been reported in patients with social phobia or high anxiety^8^,^39^. Consistently with results from previous fMRI studies, we found increased activity of the bilateral PFC in the P3b in this study, which suggests that excessive ventral attentional resources were allocated to the target stimulus and a pathological pattern was linked with too much arousal.

In the distracter condition, attenuated P3a response of the left PrCS in the anxiety patients suggests fewer dorsolateral frontoparietal attentional resources allocated to the distracter. Because of the importance of the PrCS for top-down attentional selection^40^,^41^, decreased activity of the left PrCS and wide-spread decreased group-level activity in the anxiety group reflect a decline in the top-down process in which attention is disengaged from the target-standard discrimination and reallocated to the distracter stimulus.

The anxiety group showed an anxiety-related pattern of hyperactive ventral attention networks, indicating increased stimulus-driven attention to task-relevant stimuli. Decreased functioning of the dorsolateral frontoparietal attention network implicates a decline in the top-down attention process toward task-irrelevant stimulus.

### Source characteristics of depressive disorder alone

In the depression group, decreased activity of the right PPC and right PrCS was observed during the late segment of the P3b interval. Because these areas of the dorsolateral frontoparietal attention network play an important role in goal-directed attention selection^27^,^42^, this dorsolateral hypoactivity suggests that depression has effects on the goal-directed (top-down) attention process. Depressed patients also showed decreased activity of the right INS during the late segment of the P3b interval (Table 5; Fig. 3f). As part of the salience network, the INS is involved in the modulation of excitability of the dorsal attention system^43^,^44^,^45^,^46^. The shorter P3b duration of the INS, PPC and PrCS in depressed patients reflects the creation of reduced arousal, which is inadequate to preserve the excitability of the dorsal attention network during the late period.

A depression-related pattern showed decreased functioning of the salience and dorsolateral frontoparietal attention network lateralized to the right hemisphere, which supports the assumption made in previous behavioral and EEG studies^26^,^30^,^47^,^48^ that depression might engage arousal and vigilance mechanisms lateralized to the right hemisphere. This finding also supports the valence-arousal hypothesis that depression correlates with decreased activity in the right parieto-temporal brain region associated with arousal properties^17^.

In the distracter condition, depressed patients showed decreased nSW activity in the left PrCS, which indicated a longer P3a duration. The longer P300 duration in depressed patients compared with healthy subjects indicates enhanced top-down attention processing toward the distracter during the late processing period because the ERP duration indexes the cumulative amount of underlying neural activity.

### Source characteristics of comorbidity

**Figure 5:**
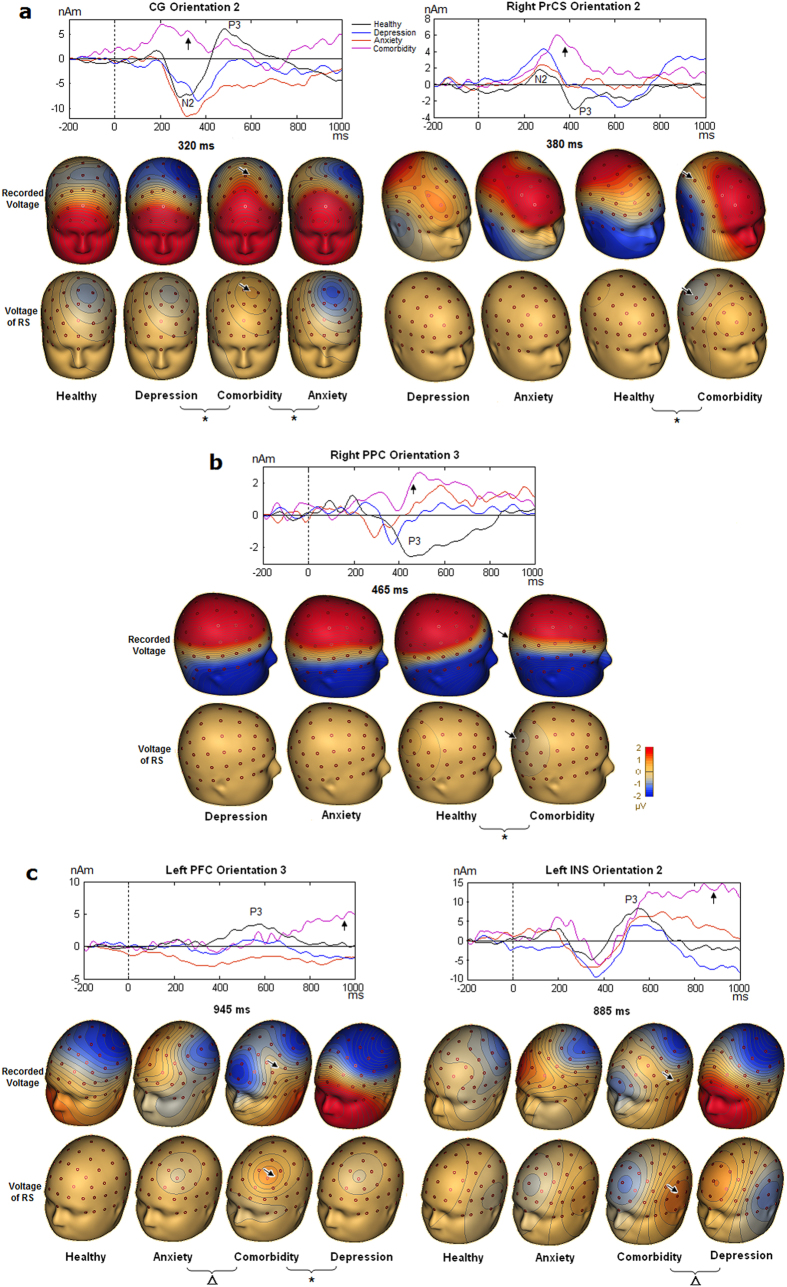
Source activities associated with the comorbidity during target stimulus processing. (**a**) Decreased activities of the CG and the right PrCS in the comorbid patients; (**b**) Decreased activity of the right PPC in the comorbid patients; (**c**). Increased activities of the left PFC and the left INS in the comorbid patients.For each plot of RS, the upper row of maps illustrates topographical maps of scalp voltage at respective latency while the lower row shows topographical maps of scalp voltage of the current flow direction of the corresponding RS. The arrow signs in the upper and lower rows of maps show the correspondence between comorbidity-related alteration of topographical map and significantly altered source activity in the comorbid patients. Asterisk * indicates significant difference with a control of family-wise error rate at level 0.05, and triangle Δ indicates significant difference with a control of family-wise error rate at level 0.1.

The comorbid patients showed significantly increased P3b activity of the left PFC (Fig. 3g) and decreased P3b activity of the right PrCS and right PPC (Fig. 5a,b). Hyperactivity of the PFC reflects a stimulus-driven anxiety-related pattern, whereas reduced functioning of the right dorsolateral frontoparietal areas reflects the depression-related hypofunction pattern.

In the CG, which is a region in the salience network, the comorbid patients showed significantly reduced N200 activity compared with those with either depression or anxiety alone (see N200/P3b complex in Fig. 5a). Because target-related CG activation was interpreted as the initiation of the task-related motor response to targets^49^, decreased CG functioning in the comorbid patients suggests inadequate motor response initiation, which entails the prolonged RT.

During the late segment of the P3b in the comorbid patients (Fig. 5c), enhanced activities of the left PFC and left INS reflect increased functioning of the ventral attention and salience networks compared with the patients with either depression or anxiety alone. Because the INS is important for both stimulus salience and task performance evaluation^45^,^50^, this later hyperactivity of the INS and PFC may reflect increased arousal allocation induced by longer RT and making more errors in the comorbid patients.

**Figure 6:**
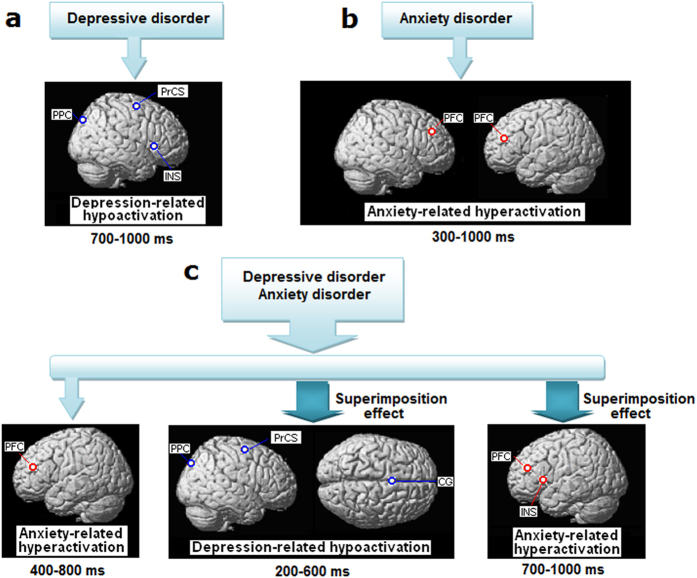
Graphic representations of depression-related and anxiety-related patterns of cortical activity and their superimposition effect in the comorbid patients. (**a**) Decreased functioning of dorsolateral frontoparietal attention network and salience network in patients having depression alone. (**b**) Stimulus-driven network hyperfunction in patients having anxiety disorders alone. (**c**) Comorbidity is characterized by aggravated hypofunction of dorsolateral frontoparietal attention network and salience network respectively engaged in goal-directed attention selection and response initiation, as well as a complex of stimulus-driven and task performance-induced network hyperfunction.

Based on the above findings, the comorbid patients showed a particular hypoactive pattern during their altered target processing (Fig. 6). Decreased functioning of the right-lateralized dorsolateral frontoparietal cortices was found during the segment of maximal P3b deflection, and decreased functioning of the CG was identified during the N200. In addition, the comorbid group was the only group to show declines in task performance. Decreased functioning of the dorsolateral frontoparietal cortices and CG in the comorbid patients reflects declined goal-directed attention and response initiation^27^,^42^,^49^, which might produce the poorer performance. Consequently, the dorsolateral hypoactivity during the main phase of the N200/P3b complex in the comorbid patients is more severe than that during the late segment of the P3b in the depression patients, despite their comparable depression severity quantified by the HRSD 17. These results suggest that the superimposition of anxiety and depression aggravated the hypofunction of the networks engaged in goal-directed attention selection and response initiation.

The comorbid patients also showed a unique hyperactive pattern (Fig. 6). In particular, hyperactive left PFC was observed during the segment of maximal P3b deflection. In addition to this earlier stimulus-driven hyperfunction, the comorbidity resulted in increased activity of the left ventral attention and salience networks during the late segment of the P3b, which increased the complexity and severity of the left-lateralized network hyperfunction. During the distracter processing, the comorbid patients also showed increased P3a activity in the left PrCS (Fig. 4c,e). This dorsolateral hyperfunction pattern indicated enhanced top-down attention processing toward the distracter. Consequently, we suggest that multiple mechanisms of network hyperfunction exist in comorbid patients, including stimulus-driven hyperfunction, task performance-induced hyperfunction involving ventral attention and salience networks, and top-down regulated hyperfunction involving the dorsal attention network. These hyperactivities are largely left-lateralized.

In summary, the superimposition effect was observed in the anxiety and depression comorbidity, and the hyperfunction involving more brain networks and the aggravated network hypofunction make up a more serious network dysfunction, which increases the difficulty of diagnosis and treatment. We further suggest that the complicated network hyperfunction in comorbidity might be linked with or caused by the aggravation of network hypofunction engaged in goal-directed attention and response. Therefore, to relieve network hyperfunction, measures should be taken to alleviate the aggravated dorsolateral network hypofunction.

### Limitation

In this study, the spatial constraints in ERP source analysis were performed with reported normal data. A limitation of the present study is that the spatial constraints were not precise due to the lack of a BOLD template from patients with depressive and anxiety disorders. However, activations in the ventral frontoparietal network (PFC,IPL,STS), dorsolateral frontoparietal attention network (PrCS, PPC), salience network (INS, CG) and IT, were generally accepted to be indispensable to the performance of the visual oddball task in both healthy and psychiatric subjects^51^,^52^. Moreover, the RS modelling does not need a precise location because of its integrative nature, which can model activity of multiple gray matter patches in its vicinity and the insensitiveness of source waveform to location error of less than 2 cm^33^. Hence, the RS modelling with the spatial constraints based on the data from healthy adults still allows for estimating psychiatric-related electrical activity in these cortical areas across patients. These RS results also showed good correspondence with the ERP ANOVA (Subsection **ERPs analysis**).

Another limitation in this study is that we used the syndromic definition of comorbid depression and anxiety, i.e., depressive disorder plus a co-existing Axis I anxiety disorder, though there are also other ways of defining comorbid depression and anxiety. Although we can extrapolate the function of certain hypo- and hyperactive brain areas in this study, we still cannot implicate these areas in certain functions, especially with small sample sizes. In the future, it will be important to confirm the results with a larger sample size and multimodal imaging data. Additionally, elderly patients with depression/anxiety should be investigated in future work to understand attentional deficits in people with late life depression/anxiety.

## Conclusion

In a visual three-stimulus oddball paradigm, an anxiety-related pattern characterized by overactive PFC was found in the anxiety group, and a depression-related pattern of reduced functioning of the right-lateralized dorsolateral frontoparietal attention network and salience network was identified in the depression group, during the late period of P300. In the comorbid patients, both anxiety- and depression-related patterns were observed. More importantly, the superimposition effect of depression and anxiety was found with: (1) aggravated network hypofunction of goal-directed attention selection and response initiation; and (2) a complex of stimulus-driven, task performance-induced and top-down regulated left-lateralized network hyperfunction. This may explain the difficulty in diagnosis and treatment of comorbidity.

This finding suggests the identification of the superimposition effect and network dysfunction be an electrophysiological biomarker for the precise diagnosis and prognosis of comorbidity. It can also help assess the effectiveness of therapeutic strategies for comorbidity.

## Additional Information

**How to cite this article**: Li, Y. *et al.* Source analysis of P3a and P3b components to investigate interaction of depression and anxiety in attentional systems. *Sci. Rep.* **5**, 17138; doi: 10.1038/srep17138 (2015).

## Supplementary Material

Supplemental Figure S1

## Figures and Tables

**Table 1:** Characteristics of participants.

		Healthy controls (n = 29)	Depressive disorder alone (n = 24)	Anxiety disorders alone (n = 22)	Comorbidity (n = 20)
Gender	Male	14	10	10	8
	Female	15	14	12	12
Age (years)	Mean	30	31.9	33.2	33.4
	SD	7.5	8.2	7.1	9.0
Education level (years)	Mean	15.7	14.8	15.6	14.5
	SD	2.2	2.3	2.0	2.8
HRSD 17	Mean	1.4	24.7	11.0	25.6
	SD	1.7	4.5	3.5	2.7
HARS	Mean	4.7	12.5	27.0	25.8
	SD	1.3	5.1	6.2	3.5

The severity of depression and anxiety was quantified by using HRSD 17 and HARS. HRSD 17 = Hamilton Rating Scale for Depression (17 items); HARS = Hamilton Anxiety Rating Scale. There were no significant differences in age or education level across groups (P > 0.05), and all groups had equivalent gender distribution.

**Table 2:** Stimulus characteristics.

Stimulus	Rate of occurrence	Task type	
		Square task (viewing angle)	Circle task (viewing angle)
Distracter	5%	1.36° ●	1.53° 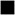
Target	5%	1.38° 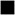	1.21° ●
Standard	90%	1.53° 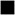	1.36° ●

Rate of occurrence, shape, and visual angle of stimuli for the visual three-stimulus oddball tasks. In the experiment, stimuli were presented in blue. The shape sizes are relative and presented for illustrative purposes

**Table 3:** Talairach coordinates of the regional sources and the residual variance (RV) of this RS model.

Regional source	Hemisphere	Talairach coordinate (mm)		
		X	Y	Z
IPL	Left	−47	−45	43
	Right	46	−49	47
PPC	Left	−30	−73	44
	Right	30	−74	44
IT	Left	−43	−61	−17
	Right	48	−57	−10
PrCS	Left	−45	0	50
	Right	46	4	53
INS	Left	−36	18	15
	Right	38	21	12
PFC	Left	−39	42	39
	Right	40	43	36
CG		−1	3	56
STS	Right	52	−44	14

The statistics of the RV for four groups in target condition are as follows. Healthy controls: 1.5% at the group level and 2.45 ± 1.1% (mean ± SD) at the single-subject level; Depression group: 2.2% at the group level and 3.13 ± 1.33% at the single-subject level; Anxiety disorders group: 2.6% at the group level and 2.86 ± 1.34% at the single-subject level; Comorbid group: 1.4% at the group level and 2.56 ± 1.2% at the single-subject level. The statistics of the RV for four groups in distracter condition are as follows. Healthy controls: 1.6% at the group level and 2.48 ± 1.12% at the single-subject level; Depression group: 1.8% at the group level and 3.23 ± 1.44% at the single-subject level; Anxiety disorders group: 1.7% at the group level and 2.97 ± 1.32% at the single-subject level; Comorbid group: 1.2% at the group level and 2.67 ± 1.4% at the single-subject level. Note that the RS model explained the scalp distribution of ERP within the time interval from 0 ms to 1000 ms, and the RV did not differ significantly among groups (F = 1.53, P = 0.21 in the target condition; F = 1.61, P = 0.19 in the distracter condition). Abbreviation: PPC: posterior parietal cortex; IPL: inferior parietal lobe; IT: inferior temporal cortex; PrCS: precentral sulcus; PFC: prefrontal cortex; INS: anterior insula; STS: superior temporal sulcus; CG: cingulate gyrus.

**Table 4:** Mean reaction time and performance rates of the four groups in visual oddball paradigm.

	Healthy control	Depression alone	Anxiety alone	Comorbidity
Reaction time (ms)	595.1	595.9	581.1	652.3*
Target hit rate	83.2%	82.9%	85.6%	77.8%*
False positive rate (standard)	0.88%	2.03%	1.26%	2.86%*
False positive rate (distracter)	0.56%	1.01%	1.27%	0.86%

^*^p < 0.05, in comparison with control group.

**Table 5:** Characteristics of significant clusters in the target and distracter conditions.

Condition	Group comparison	Regional source	Orientation	Time-range (ms)	Unadjusted P-value
Target	D vs H	Right PPC	1	672–968	0.015 Δ*
		Right PrCS	3	685–1000	0.008 Δ*
		Right INS	3	690–1000	0.047
	A vs H	Left PFC	1	325–1000	0.007 Δ*
		Right PFC	1	268–1000	0.023 Δ
	C vs H	Right PrCS	2	305–495	0.015 Δ*
		Left PFC	1	402–815	0.039 Δ*
		Right PPC	3	410–625	0.048 Δ*
	A vs D	Left PFC	1	610–1000	0.019 Δ
		Right INS	3	690– 1000	0.010 Δ*
	C vs A	CG	2	195–443	0.003 Δ*
		Left PFC	3	775–1000	0.010 Δ
	C vs D	CG	2	190–400	0.018 Δ*
		Left PFC	3	760–1000	0.021 Δ*
		Left INS	2	670–1000	0.040 Δ
Distracter	D vs H	Left PrCS	1	512–888	0.039 Δ
	A vs H	Left PrCS	1	368–494	0.030 Δ
	C vs H	Left PrCS	2	305–490	0.012 Δ
	A vs D	Left PrCS	1	256–525	0.017 Δ
	C vs A	Left PrCS	2	263–497	0.010 Δ
	C vs D	Right STS	1	902–1000	0.048 Δ

Note: the depression disorder alone, anxiety disorders alone, comorbid patients and healthy controls are respectively abbreviated as D, A, C and H. Asterisk * indicates significant difference with a control of family-wise error rate at level 0.05, triangle Δ indicates significant difference with a control of family-wise error rate at level 0.1 (significant on a trend level).
