# Supplementary material for: Source analysis of P3a and P3b components to investigate interaction of depression and anxiety in attentional systems
Source: Sci Rep. 2015 Nov 24;5:17138. doi: 10.1038/srep17138 (PMC4657106; doi:10.1038/srep17138)
Supplement: Supplemental Figure S1 [file srep17138-s1.doc]

**Source analysis of P3a and P3b components to investigate interaction of depression and anxiety in attentional systems**

Yuezhi Li, Wuyi Wang, Tiebang Liu, Lijie Ren, Yunfei Zhou, Changhong Yu, Xingda Qu &Yong Hu

Y

Acquisition of the 6 arrays of P values for significant clusters by permutation test between any two groups from four participant groups

Sequential comparison was finished without stop? Or the stopped i was unchanged after any renewal of P(i)?

End

output:

Sort the highest P value of each array as P(i) (i=1,2,...,6), i.e. P(1)<P(2)<……<P(5)<P(6)

For a significance level of α = 0.05 or 0.10 (significance on a trend level), each P(i) was sequentially compared with α/(7–i), starting from i = 1. The sequential comparison was stopped when P(i) > α/(7-i)

Remove one of elements {P(i)~P(6)} from its array so that it will be replaced by a minimum P value after renewal of {P(i)~P(6)} in a next cycle

N

**Supplemental Figure S1** Flow chart of Holm-Bonferroni procedure.
